# How to Make a Dolphin: Molecular Signature of Positive Selection in Cetacean Genome

**DOI:** 10.1371/journal.pone.0065491

**Published:** 2013-06-19

**Authors:** Mariana F. Nery, Dimar J. González, Juan C. Opazo

**Affiliations:** 1 Instituto de Ciencias Ambientales y Evolutivas, Facultad de Ciencias, Universidad Austral de Chile, Valdivia, Chile; 2 Programa de Doctorado en Ciencias mención Ecología y Evolución, Facultad de Ciencias, Universidad Austral de Chile, Valdivia, Chile; Swiss Federal Institute of Technology (ETH Zurich), Switzerland

## Abstract

Cetaceans are unique in being the only mammals completely adapted to an aquatic environment. This adaptation has required complex changes and sometimes a complete restructuring of physiology, behavior and morphology. Identifying genes that have been subjected to selection pressure during cetacean evolution would greatly enhance our knowledge of the ways in which genetic variation in this mammalian order has been shaped by natural selection. Here, we performed a genome-wide scan for positive selection in the dolphin lineage. We employed models of codon substitution that account for variation of selective pressure over branches on the tree and across sites in a sequence. We analyzed 7,859 nuclear-coding ortholog genes and using a series of likelihood ratio tests (LRTs), we identified 376 genes (4.8%) with molecular signatures of positive selection in the dolphin lineage. We used the cow as the sister group and compared estimates of selection in the cetacean genome to this using the same methods. This allowed us to define which genes have been exclusively under positive selection in the dolphin lineage. The enrichment analysis found that the identified positively selected genes are significantly over-represented for three exclusive functional categories only in the dolphin lineage: segment specification, mesoderm development and system development. Of particular interest for cetacean adaptation to an aquatic life are the following GeneOntology targets under positive selection: genes related to kidney, heart, lung, eye, ear and nervous system development.

## Introduction

A central goal in evolutionary biology is to understand the relative contribution of the different evolutionary forces in the origin of new species. The answer to this long-standing question has often been framed in terms of the relative contribution of natural selection to extant patterns of genetic variation. In particular, there has been great interest in detecting genes or genomic regions under positive selection since these provide evidence for adaptive changes in protein function. Identifying genes with a signature of adaptive evolution could shed light on the type of genetic variation that contributes to the origin of phenotypic diversity, and to which extent positive selection plays a major role in evolutionary change.

With the increasing availability of genome sequences and sophisticated analytical tools, the interest in finding targets of positive selection has increased in the last decade. In mammals most of the studies are focused on understanding the genetic changes that occurred during human ancestry, and have identified several genes that show strong evidence of positive selection, including those related to immunity, sensory perception, reproduction and host-pathogen interactions [1]–[7].

From all known mammalian lineages, the evolutionary history of cetaceans is one of the most eloquent examples of extensive adaptations to meet new environmental conditions [8]. The macroevolutionary transition from a fully terrestrial quadruped to an obligate aquatic form involved extensive changes in the morphological, physiological and behavioral systems [9]. These changes likely resulted from selective pressures for new genotypes that were better suited to the novel environments. Paleontological evidence indicates a series of transformations from more terrestrial to more aquatic lifestyle as we move from the most ancestral (represented by the *Pakicetus* from the early Eocene of Pakistan, about 50 million years ago [9]) to the most recent species. These transitions have affected most of the cetacean biological systems and allowed them to diversify to different aquatic habitats, dispersing across the world's oceans, and into estuaries and even rivers [10].

Whereas cetacean evolution has been substantially elucidated by a fossil record accumulated for the past two decades [11]–[14], there is a substantial lack of information regarding the evolutionary forces and mechanisms that underlay the transition from a terrestrial habitat to a fully aquatic life. Identifying genes that have been targets of positive selection in the cetacean lineage can provide important insights into their evolutionary history. There are a number of recent efforts reporting individual genes that show signals of selection in cetaceans [15]–[21]. Nevertheless, these examples have all been discovered in studies of candidate genes where there was an *a priori* hypothesis of selection. Still, very little is known about how widespread such signals are. A genome-wide scan for those signatures is the first and necessary step toward gaining a comprehensive and systematic understanding of the evolution of this unique mammalian order composed by only aquatic animals.

The aim of this work was to perform a genome-wide scan for genes that have been targeted by positive Darwinian selection on the dolphin lineage. To accomplish this goal we have searched for the most complete set of known 1∶1 orthologs in different mammalian species, and used statistical methods to test the potential role of positive selection on the dolphin lineage. Here we report that several genes have been under the action of positive selection and that most of them are categorized in overrepresented functional classes such as segment specification and development.

## Materials and Methods

### Orthology

Orthologous relationships were obtained from the Orthologous Matrix (OMA) project [22]–[23]. The OMA project is a large-scale effort to identify groups of orthologous genes in publicly available genomes in order to define groups of single-copy 1∶1 orthologs that are used to define the different OMA groups. The criteria used to establish the membership of the different OMA groups are very conservative since a careful search for possibly paralogy discards all suspicious matches [22], [24]. As a result, the OMA project has proved to be more accurate and offered the best performance when compared to other methods of large-scale ortholog determination [23].

**Figure 1:**
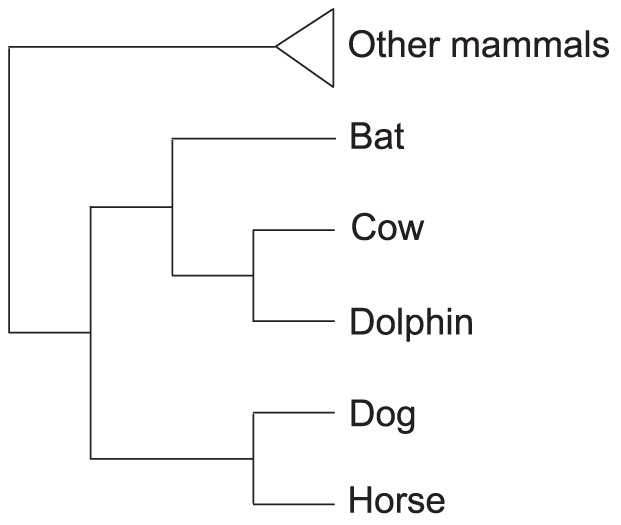
Tree topology used for the analysis of the natural selection in which the five laurasiatherian species included in all the orthologous groups are depicted. This tree is based on published literature [57]–[59].

In order to maximize the number of orthologous genes to be analyzed, and to account for the minimum number of species to perform a maximum likelihood analysis of natural selection, we developed an in-house program that performs what we call a “non-exact search of orthologous groups”. In a non-exact search, the user is able to define the identity of the species that must be present in each group, and also the minimum number of species per group. The unspecified species are restricted to a wider taxonomical category, which is also specified by the user (*e.g.* mammals). The choice of the species that must be present in each orthologous group included in the study, and the wider taxonomical category from which the unspecified species come from, should be oriented to the main goal of the study. As our goal was to investigate the potential role of positive selection on the dolphin lineage, we defined a set of five laurasiatherian species (bottlenose dolphin *Tursiops truncatus*; cow, *Bos taurus*; bat, Myotis *lucifugus*; dog, *Canis familiaris*; horse, *Equus caballus*) that were present in all orthologous groups (Figure 1). Throughout the text we use the term ‘dolphin’ to refer to the ‘bottlenose dolphin’ species (*T. truncatus*). We also constrained our search to a minimum of 10 species per group, from which only mammals were included. Finally, and depending on the species composition of each orthologous group, the program generates a unique phylogenetic tree, comprised of the ortholog specific set of species, based on the most recent phylogenetic hypothesis, and performs the natural selection analysis. Nucleotide translated sequences were aligned using MUSCLE [25], and nucleotide alignments were generated using the amino acid alignments as a template with the software PAL2NAL [26].

### Natural selection analysis

In order to investigate the possible role of positive selection on the dolphin lineage, we explored variation in ω, the ratio of the rate of non-synonymous substitutions (*d_N_*) to the rate of synonymous substitutions (*d_S_*), in a maximum likelihood framework using the codeml program from PAML v4.4 [27]. Briefly, if amino acid replacements are neutral, then *d_N_* and *d_S_* would be very similar and ω = *d_N_*/*d_S_*≈1. In contrast, under purifying selection most amino acid substitutions would be deleterious, and so ω<1, whereas under positive selection amino acid replacements would be advantageous, and so ω>1. We applied branch-site models, which explore changes in ω for a set of sites in a specific branch of the tree in order to assess changes in their selective regime [28]. In this case the dolphin terminal branch was labeled as the foreground branch in each phylogenetic tree generated for each orthologous group.

It is important to note that we tested unique and independent hypotheses for each ortholog, since each orthologous gene has a distinct species composition (*i.e.* always with the five laurasiatherians mentioned before – dolphin, cow, horse, dog and bat – plus at least five additional mammalian species). We compared the modified model A [28]–[29], in which selection at some sites is allowed to change to ω>1 in the foreground branch, with the corresponding null hypothesis in which no such changes are allowed. To make our results comparable we repeated the same analysis but this time labeling the cow terminal branch, which in our phylogenetic design is the sister group species of dolphin, as a foreground branch. This approach allowed us to define which genes have been exclusively under positive selection in the dolphin lineage. In all cases, three starting ω values (0.5, 1 and 2) were used to check the existence of multiple local optima, and the run with the best likelihood score was used for further analyses. Nested models were compared using the likelihood ratio test (LRT), and the significance was established at p<0.05. Although the current release of the bottlenose dolphin genome has only 2.59% coverage, [30] and [31] found few sequencing errors in the dolphin genes, and predicted an error of approximately 0.9 [31] and 1.5 [30] bases per 1000 bases. Therefore, the dolphin genome has enough quality to ensure reliable results regarding the detection of positive selection.

### Functional analysis

To gain insight on the putative functions of the positively selected genes (PSGs) found on the dolphin lineage, we performed a functional annotation using the Gene Ontology terminology [32] according to the web-based program CateGOrizer [33]. Additionally, to determine if any category showed statistically overall greater evidence for positive selection, we performed a Gene Ontology enrichment analysis by using the binomial statistic test implemented in Panther database [34]. The enrichment analysis identifies PSGs whose abundance is significantly different between two sets of annotated genes, in our particular case PSGs dataset against a reference of all genes analyzed. We used the Bonferroni correction for multiple testing as implemented on Panther database, and a p-value cutoff of 0.05.

## Results

**Figure 2:**
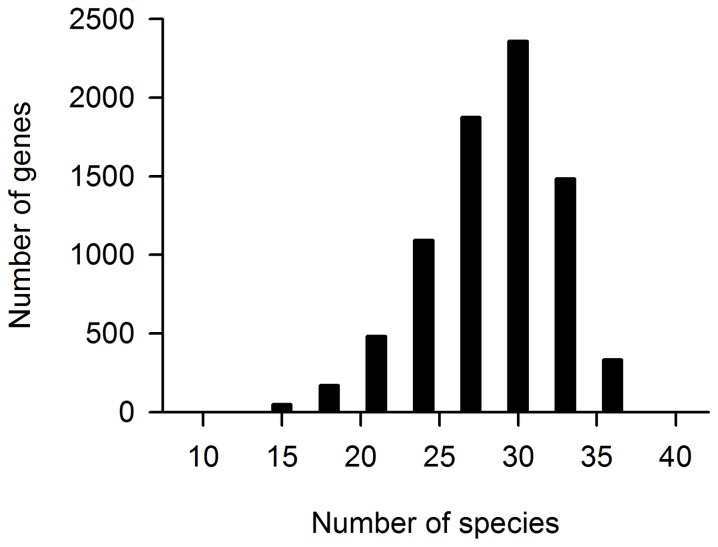
Distribution of the number of species included in each orthologous group.

To investigate the impact of positive Darwinian selection in dolphin evolution, we analyzed a conservative set of unambiguous 7,859 single copy 1∶1 orthologs. The number of species per ortholog groups was normally distributed (Fig. 2), the great majority of groups (5,720) contained between 27 to 33 species (Fig. 2). The five laurasiatherian species (bottlenose dolphin, cow, bat, horse and dog) were always present. In our first analysis, where the dolphin terminal branch was labeled as a foreground branch, we identified 376 (4.8%) genes under positive selection, whereas when the cow terminal branch was labeled as foreground branch we found 448 (5.7%) positively selected genes (Table S1).

**Figure 3:**
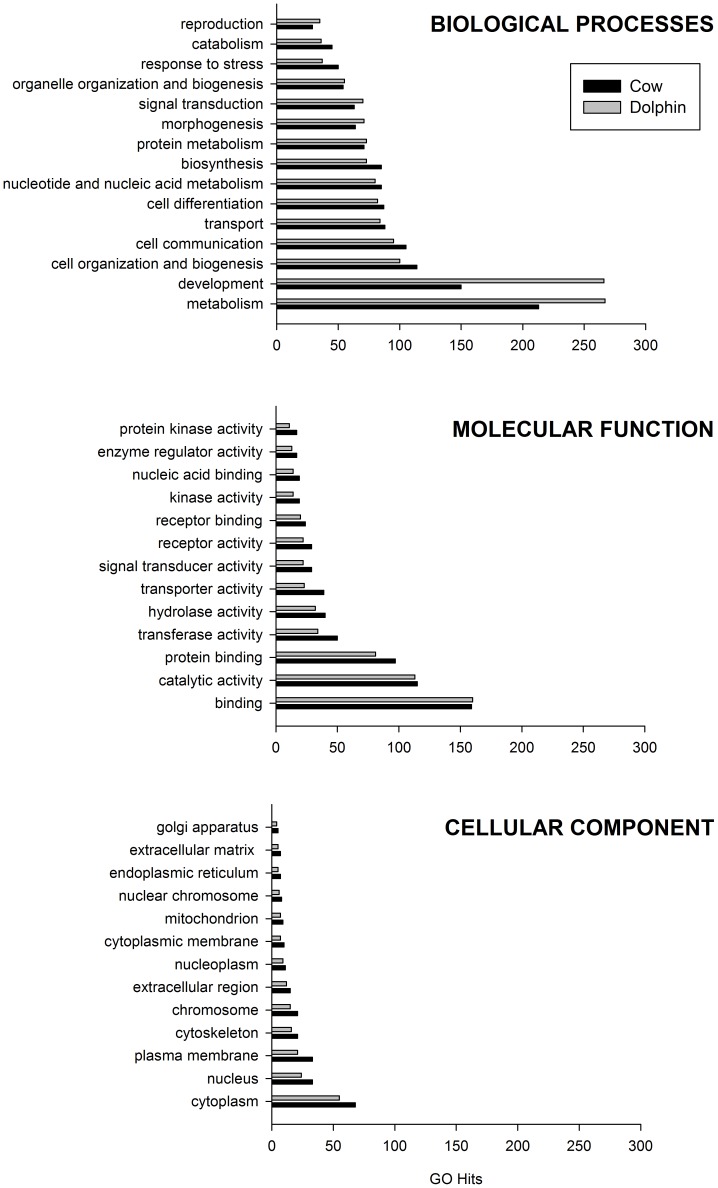
GO distribution in the three gene ontology domains (biological processes, molecular function and cellular component) among positively selected genes. Only the first 15 categories from each domain are shown. PSG, positively selected genes.

To understand the biological significance of the PSGs we used the program CateGOrizer to classify them according to the scheme established by the Gene Ontology project (Fig. 3). This analysis revealed the most GO hits in ‘biological process’ (Fig. 3) with significantly fewer hits in ‘molecular function’ and ‘cellular component’ (Fig. 3). Within the ‘biological process’ category, most gene classes were distributed similarly between dolphin and cow, with the exception of development and metabolism, in which the dolphin lineage had considerable more GO hits in comparison to the cow (Fig. 3).

**Figure 4:**
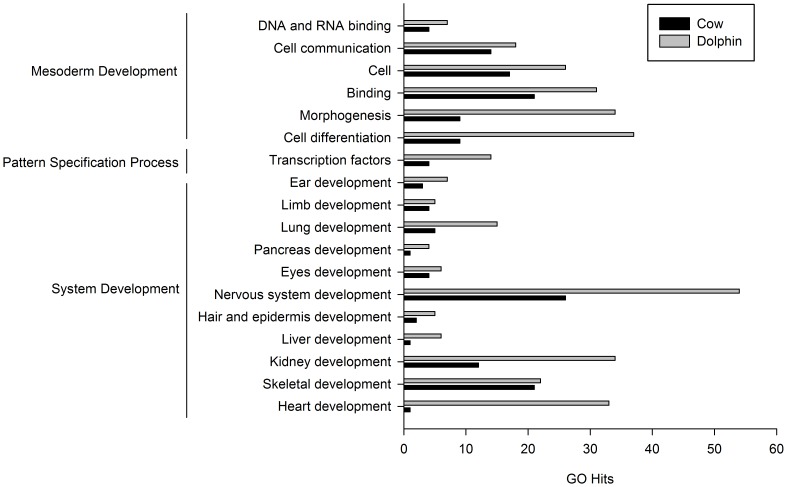
GO distribution within the functional categories overrepresented in the dolphin lineage.

To test whether specific categories of genes were statistically under or overrepresented among the PSGs, we performed an enrichment analysis using the Panther classification system (Table 1). According to this analysis we identified classes of genes showing an excess of PSGs only in the developmental process category, which is nested within the biological process domain, in the dolphin lineage (Table 1). The overrepresented categories were mesoderm development, pattern specification process and system development (Table 1). To gain further insight into the biological meaning of this result, we identified less inclusive categories within those overrepresented categories in both tested lineages. As shown in figure 4, the most impacted categories were the development of the nervous system, kidney, heart and skeletal muscle in the system development category, whereas in the mesoderm development category were cell differentiation, morphogenesis, binding and cell the most impacted categories (Fig. 4).

**Table 1:** Enrichment analysis of positively selected genes in the dolphin and cow lineages.

GO term	PANTHER category	All genes	Bottlenose dolphin			Cow		
			PSG	PSG E	p-value	PSG	PSG E	p-value
	Biological Processes							
	Developmental process							
GO:0007498	Mesoderm development	337	34	15.9	0.0058	22	19.3	1
GO:0007389	Pattern specification process	77	15	3.64	0.0009	9	4.4	1
GO:0048731	System development	497	47	23.5	0.0009	36	28.4	1

## Discussion

There are several studies that have looked for the role of positive Darwinian selection at the genome level [1], [2], [6], [7], [35]–[38]. Among them, mammals are the vertebrate group that has attracted most of the attention, with a special emphasis in humans and other primates. Results from these studies vary in the proportion of genes showing signatures of positive selection. For example, [6] and [36] estimated a proportion of genes under positive selection in the human lineage of 0.8 and 1.1%, respectively, whereas other authors have estimated proportions between 2.4 to 9% [1], [2], [7], [35]. In non-model species there is much less evidence, the numbers are also variable and difficult to compare as different studies report results for different species [7], [35], [37]. In one of the few studies focusing in a non-model mammal, [37] analyzed 7,164 nuclear-encoded genes to identify the general background of positive selection on the bat lineage and they found 72 genes (1.005%) showing evidence of positive selection. On the other hand, [35] estimated that 4.9% of the tested genes are under the action of positive selection in the pig lineage, a species that belong to the same mammalian order (Cetartiodactyla) as the model species used here. Very recently, [30] and [31] published the results of a genome scan analysis of the dolphin genome. The first study [30] discovered 2.26% of genes potentially under positive selection, whereas [31] found a total of 3.1% of the genes identified as having undergone positive selection.

In our study, we were able to identify 4.8% of nuclear-encoded genes showing evidence of positive selection in the dolphin lineage. Although these numbers are similar to the reported values by other studies, especially when compared to more closely related species [35], we caution that the use of different phylogenetic designs among the studies make comparisons difficult. It is important to highlight that the use of different taxonomic samplings (numbers and identity of the species), alignment methods, orthology predictions, among others, make comparisons very difficult from different studies. We believe our study uses a conservative and reliable set of methods to attain results and allow comparison: a conservative way to define orthology, and an appropriate number of species per orthologous group (Fig. 2) in order to meet statistical criteria established by the software authors [39], [40]. The importance of the methods we used is revealed by comparing our study with the two previous genome-scans of the dolphin genome [30], [31]. A comparison of the gene lists analyzed by [30], [31] and this study, reveal a high degree of overlap that varies from 55 to 83%. However, this overlap almost disappears when the lists of genes inferred under the action of positive selection are compared (from 3.2 to 8.7%). It means that by analyzing almost the same set of genes, the three studies ultimately found distinct results.

In our study, the proportion of genes identified under the action of natural selection in the dolphin and cow lineages (4.8 and 5.7% respectively) show no important differences regarding the prevalence of selection as a force acting at the genome level. Moreover, in both lineages, positive selection appears to have affected a wide variety of classes of genes instead of acting on few ones (Fig. 3). This pattern of widespread signatures was described before in other genome-wide studies [1], [2], [6], [7], [36], [41], [42]. However, in comparison with these previous studies, which are mostly focused on primate species (especially humans), and also with the recent genome-scans of dolphin genome [30], [31], we found no evidence for enrichment of the immunity, sensory perception and sexual reproduction functional categories in neither dolphin or cow lineages (Table 1 and Fig. 4). Instead, we found that the system development, pattern specification process and mesoderm development functional categories – all linked to developmental process in general – are significantly over-represented only in the dolphin lineage. Although we were only able to include a single dolphin species, these enriched classes of PSGs are likely to reflect the process of adaptation to the new conditions that cetaceans in general had to face on their early evolution.

The GO category “pattern specification” is defined as the processes in which segments assume individual identities. This category is overrepresented among the PSGs in the dolphin lineage and includes such genes as MET, FOXP2, TRIM63, FOXO3, CD2 and PTCH1. Changes in such genes are an important indication of cetacean evolution because they have been suggested as potential causes of morphological evolution, and there is evidence that changes in transcription factors expression patterns and in protein function contributed to a variety of small and large morphological changes during metazoan evolutionary history [43]. It is likely that homeotic genes may have played a key role in aquatic mammals body plan development. Indeed, [17] reported that the ratio of nonsynonymous to synonymous substitutions of 5′HoxD genes was approximately three times higher in cetaceans when compared to other terrestrial mammals. Their results provide evidence of strong selection, and reject neutral evolution, on the branch leading to cetaceans for this specific homeotic gene [17].

We also suggest that it is not surprising that the system development and mesoderm development functional categories also appear as overrepresented in the dolphin lineage, since it is largely known that marine mammals show many deviations from the typical terrestrial mammalian characteristics, and most of these unique features emerge during development. Among the genes related to the system development category, some of them are of particular interest for the dolphin lineage. For example, we found almost three-times higher GOs related to kidney development to be under positive Darwinian selection in the dolphin lineage, when compared to the cow lineage (genes such SMAD1, NPNT, LEF1, SERPINF1 and AQP2). In this regard it has been reported that whales and dolphins are able to concentrate the urine to an osmolarity greater than the sea water [44]. Additionally, it is well known that dolphins possess reticulate kidneys similar to those of otters and bear (also aquatic mammals), but it is believed that the reticulate kidneys of cetaceans probably evolved in response to their large body and diving abilities rather to the osmotic challenge posed by an aquatic environment [45]–[47].

Also, those GOs related to the heart development were found to be in the most striking higher proportion in comparison to the cow (33-times higher). The dolphin heart shows specific features that are likely to be diving adaptations, such as anastomoses between the dorsal and the ventral inter ventricular arteries, and hypertrophy of the right ventricle [48]. Examples of positively selected genes related to heart development are ADAM9, NKX2, CAD15, CRFR2, GDF9, CADH3, TAB2 and PLN.

Another organ very represented among developmental PSGs in the dolphin lineage is the lung (three-times more GOs related to lung development in dolphin than in cow genome; genes such RSPO2, LEF1 and FOXP2 are related to lung morphogenesis and development). Cetaceans show the most modified lung among any other mammal: an increased cartilaginous support, reinforcement of peripheral airways, lost of respiratory bronchioles, and presence of bronchial sphincters, all thought to be adaptations in response to the effects of pressure during their diving behavior [49].

In addition, dolphin sensorial system had undergone extensive adaptations to an aquatic habitat, diverging remarkably from their terrestrial counterparts. Thus, as expected, several genes related to nervous system were found to be under positive selection in the dolphin lineage (MET, NPNT, RBM23, FOXP2, WWP2, WDR17, NEDD1, GRAP, IFRD1, FHL1, GCM1, SRC, CHL1, CELF2, PTPRN, THOC6, MEGF8, FOXO3, DCLK3, ROBO4). More specifically, PSGs related to the eye development were also found to occur in higher proportion in the dolphin lineage (POU4F2, NES, FOXP2). This corresponds to the well established idea that the eye of aquatic mammals shows remarkable adaptations to both underwater and aerial vision as they have developed several specific morphological and functional specializations in the optics, retina, lens and other eye structures [50].

Other GOs that were under positive selection exclusively in dolphin lineage, such as those related to the hindlimb morphogenesis (RSPO2 and PTCH1) and hair follicle development (TGM3), can also be linked to adaptation to an aquatic life. Regarding the hindlimb, the cetacean fossil record shows a progressive reduction and loss of the hindlimb and also a disassociation of the pelvic girdle from the vertebral column as they became less dependent on land and developed tail flukes [51]. Among extant cetaceans, only vestiges of the hindlimb skeleton can be found, and they are contained within the body wall [52]. With respect to the hair follicle development, one feature that is easily recognized in dolphins is the absence of hair, and hairless is a key adaptation to live in the aquatic habitat, since the lack of fur allow cetaceans to increase their hydrodynamic and subaquatic movements.

Similar to our results, the previous genome scan of dolphin genome [30] also encountered genes related to hair, lung, vision, nervous and cardiovascular system with signals of positive selection, but could not identify these genes in any enriched category when compared to other laurasiatherian lineages. In contrast to our study, and similarly to the other genome-scan analysis in dolphin genome [31], they found signatures of positive selection in several genes related to energy metabolism [30]. These differences may be due to the fact that these studies used neither the same list of genes nor the same group of species in their analyses, and it highlights the divergence in interpretations that can emerge with the use of different treatments of the genomic data.

The dolphin lineage exhibited evidence of positive selection of multiple genes that can be linked to cetacean specializations to the aquatic environment. But it is important to note that our analyses and conclusions are based on a single bottlenose dolphin genome. The question of whether the identified PSGs are related to general cetacean adaptations to the aquatic environment or represent specific adaptations of *T. truncatus* remains open. With the advent of more sequenced cetacean genomes, the next step is to investigate the pattern of positive selection among multiple cetacean species.

Our study has exclusively focused on protein coding genes, but our results cannot exclude functional evolution by regulatory mechanisms. Although few genomic or single-locus studies have examined regulatory regions, and none of them support the claim for a predominance of regulatory mutations in adaptive evolution [53]–[55], we don't discard their relevance for cetacean evolution. The functional effects of mutations in regulatory elements are still largely unknown and it remains to be established at a genome level the relative contribution of changes in gene regulation as mechanisms of evolutionary change. It is likely that adaptation and speciation proceed through a combination of both regulatory and structural changes, with substantial empirical evidence that the latter are more predominant, at least so far [56]. Genome-wide studies are a valuable first approach to understand the role of natural selection in one of the most fundamental questions in evolutionary biology, how new lineages arise. They also generate new hypotheses regarding the physiological and biochemical consequences of natural selection that can be tested using the state of the art lab protocols. In this particular case further investigation of the PSGs identified in this study will shed more light into the nature of the adaptive events that gave rise to the cetacean lineage.

It is now a general consensus that both natural selection and genetic drift play important roles in evolutionary change. The major issues today are how strong are the effects of positive selection, which genes are under positive selection, what are the consequences of this selection in terms of phenotypic adaptation, and what proportion of a given genome has been targeted by natural selection. The answer to all these questions will bring light to our understanding of speciation and the nature of adaptation. What is currently clear is that positive selection is indeed an important source of evolutionary innovation and has undoubtedly played a critical role in the origin and subsequent evolution of cetaceans.

## Supporting Information

Table S1 **List of all genes included in this study and the positively selected genes found in the dolphin and cow lineages.**
(XLSX)
